# The role of carbohydrate drinks in preoperative nutrition: comment 2

**DOI:** 10.1308/003588413X13511609956499

**Published:** 2013-01

**Authors:** WK Mitchell, JN Lund, JP Williams

**Affiliations:** University of Nottingham, Derby,UK

We read with interest the review of Jones *et al* looking at the role of CHO drinks in perioperative nutrition for elective colorectal surgery. However, we have some concerns regarding the rigour of assessment of elements of the evidence base and the authors’ accuracy in presentation of this evidence. The largest randomised controlled trial referenced (and one of only two that looked at clinical outcomes in patients undergoing colorectal surgery in the review) is that of Mathur *et al*.^1^ The review by Jones *et al* states that Mathur *et al* showed CHO supplements reduced length of hospital stay. This is at odds with Mathur’s own conclusion: ‘Preoperative CHO treatment did not improve postoperative fatigue or length of hospital stay after major abdominal surgery.’^1^

Furthermore, the work of Svanfeldt *et al* ^2^ is misquoted in this review, suggesting it showed that: ‘whole-body protein did not change in the high CHO group whereas it was more negative in the low CHO group after surgery…’ Svanfeldt *et al* investigated whole-body protein kinetics via a stable isotope labelled amino acid technique and not whole-body protein. This protein kinetic study looked at changes in protein balance before and after colorectal surgery. Whole-body protein balance was shown to be negative at a set point in time during the preoperative fast and again at a set point during the early postoperative period. The rate of loss of protein mass at this instant was faster in the group receiving low dose preoperative CHO than in the high dose group. To interpret this as meaning whole-body protein did not change in the perioperative period in the group receiving high dose CHO is erroneous.

While Yuill *et al* have shown a reduced loss of muscle mass postoperatively in patients receiving CHO,^3^ this has been in upper gastrointestinal (GI) surgery and not colorectal surgery. Other studies such as that by Mathur *et al* ^1^ *do* measure total body protein in lower GI surgery but have not as yet demonstrated that CHO can significantly attenuate the perioperative loss of total body protein.

We agree that measures to minimise the stress response to surgery will benefit our patients. Evidence exists that supports the implementation of ERAS programmes,^4^ most of which include preoperative CHO supplementation. However, it is important for us to present in context an accurate evidence base supporting each component of ERAS to maximise the benefit to the patient with the minimum number of steps in the process. The case for CHO perioperative drink is not settled and should not become established as dogma (which applies to any other step in the process) until it is proven.
